# Inhibitory Effects of Linear Lipopeptides From a Marine *Bacillus subtilis* on the Wheat Blast Fungus *Magnaporthe oryzae Triticum*

**DOI:** 10.3389/fmicb.2020.00665

**Published:** 2020-04-30

**Authors:** Moutoshi Chakraborty, Nur Uddin Mahmud, Dipali Rani Gupta, Fakir Shahidullah Tareq, Hee Jae Shin, Tofazzal Islam

**Affiliations:** ^1^Institute of Biotechnology and Genetic Engineering, Bangabandhu Sheikh Mujibur Rahman Agricultural University, Gazipur, Bangladesh; ^2^Department of Nutrition and Food Sciences, University of Maryland, College Park, College Park, MD, United States; ^3^Department of Marine Biotechnology, University of Science & Technology, Daejeon, South Korea; ^4^Marine Natural Products Chemistry Laboratory, Korea Institute of Ocean Science & Technology, Busan, South Korea

**Keywords:** antifungal secondary metabolites, abnormal appressoria, *Bacillus subtilis*, biocontrol, deformed germ tube, linear lipopeptides, *Magnaporthe oryzae Triticum*, wheat blast disease

## Abstract

Wheat blast is a devastating fungal disease caused by a filamentous fungus, *Magnaporthe oryzae Triticum* (MoT) pathotype, which poses a serious threat to food security of South America and South Asia. In the course of screening novel bioactive secondary metabolites, we found that some secondary metabolites from a marine *Bacillus subtilis* strain 109GGC020 remarkably inhibited the growth of *M. oryzae Triticum in vitro* at 20 μg/disk. We tested a number of natural compounds derived from microorganisms and plants and found that five recently discovered linear non-cytotoxic lipopeptides, gageopeptides A–D (**1–4**) and gageotetrin B (**5**) from the strain 109GGC020 inhibited the growth of MoT mycelia in a dose-dependent manner. Among the five compounds studied, gageotetrin B (**5**) displayed the highest mycelial growth inhibition of MoT followed by gageopeptide C (**3**), gageopeptide D (**4**), gageopeptide A (**1**), and gageopeptide B (**2**) with minimum inhibitory concentrations (MICs) of 1.5, 2.5, 2.5, 10.0, and 10.0 μg/disk, respectively. Application of these natural compounds has also completely blocked formation of conidia in the MoT fungal mycelia in the agar medium. Further bioassay revealed that these compounds (**1–5**) inhibited the germination of MoT conidia and, if germinated, induced deformation of germ tube and/or abnormal appressoria. Interestingly, application of these linear lipopeptides (**1–5**) significantly suppressed wheat blast disease on detached wheat leaves. This is the first report of the inhibition of mycelial growth, conidiogenesis, conidial germination, and morphological alterations in the germinated conidia and suppression of wheat blast disease by linear lipopeptides from the strain of *B. subtilis*. A further study is needed to evaluate the mode of action of these natural compounds for considering them as biopesticides for managing this notorious cereal killer.

## Introduction

Wheat (*Triticum aestivum* L.) is a critical staple food providing 20% of the calories and over 25% of the protein consumed by humans [Food and Agriculture Organization (FAO),^1^ ]. Wheat blast disease caused by a filamentous fungus *Magnaporthe oryzae Triticum* (MoT) pathotype is the most destructive fungal disease affecting wheat production in several South American countries (Ceresini et al., 2019) and was recently introduced to Bangladesh (Islam et al., 2016a). Upon first emergence in Brazil in 1985, wheat blast was spread to Paraguay, Argentina, and Bolivia within a few years (Ceresini et al., 2019). Because of the wheat blast outbreak in 2016, nearly 15,000 hectares of wheat crops was devastated, which resulted in about 15% crop loss in Bangladesh (Islam et al., 2016a; Malaker et al., 2016). Plant pathologists have predicted that this fungal disease has a high possibility of spreading to some of the world’s top 10 wheat-producing countries such as China, India, and Pakistan, ranked first, second, and seventh, respectively (Islam et al., 2016a, 2019; Kamoun et al., 2019).

Wheat blast affects wheat plants at all developmental stages and can attack leaves, stems, nodes, and panicles (Wilson and Talbot, 2009; Islam et al., 2016a, 2019; Ceresini et al., 2019). Foliar infection is triggered by attaching of a hyaline, pyriform, a three-celled conidium of MoT to the cuticle of the leaf. Through an adhesive, the spore attaches to the hydrophobic cuticle and germinates, producing a small germ tube. Then the fungus destroys the plant hosts’ intact cuticles by developing complex structures called appressoria, which are melanin-pigmented and septate structures that initially develop at the tips of germ tubes (Tufan et al., 2009; Ryder and Talbot, 2015). Appressoria creates substantial turgor that translates into physical force and forms a narrow penetration peg at the base, rupturing the cuticle and allowing entry into the epidermis of the host. Invasion of plant tissue occurs by bulbous, invasive hyphae invaginating host plasma membrane and invading epidermal cells (Tufan et al., 2009; Wilson and Talbot, 2009). Wheat blast predominantly hits spikes; it bleaches the affected spikes, resulting in either deformed seed or no seed development. The heavily affected wheat head may die, resulting in drastic yield reduction. Bleaching of spikelets and whole head at the premature stage is thus the most common identifiable symptom (Igarashi, 1990; Islam et al., 2016a, 2019). Infected seeds and airborne spores typically spread the disease, and the fungus may survive in contaminated crop residues and seeds (Urashima et al., 1999). Therefore, critical stages in the disease cycle caused by MoT include pyriform conidia produced from cylindrical conidiophores and germination of conidia with appressorial structures at the tip of germ tubes (Tufan et al., 2009). The disruption of any of these asexual life stages eliminates the possibility of pathogenesis. Discovery of natural compounds that disrupt any of these asexual life stages considers as the first step of the development of a new fungicide against the MoT.

Fungicide application and growing resistant varieties are found to be effective methods of the wheat blast disease control caused by MoT. Farmers currently depend on extensive application of commercial fungicides to protect their wheat crop. Most of the chemical fungicides are hazardous to the environment or health of living organisms including humans (Nannipieri, 1994; Suprapta, 2012). Indiscriminate use of synthetic fungicides in plant protection results in pathogenic fungicide resistance (Kohli et al., 2011; Ceresini et al., 2019). Widespread resistance to fungicides limits their effectiveness, for example, extensive use of strobilurin (QoI) and triazole fungicides in Brazil has resulted in widespread distribution of *cyt b*, *Pp*, *Pg*, *Pu*, and *Pygt* mutants that are resistant to isolated strains from wheat and other poaceous species (Castroagudín et al., 2015; Dorigan et al., 2019). Moreover, antifungal resistance and toxicity issues of conventional fungicides illustrate the need to search for new antifungal agents to enable development of environmentally suitable alternative fungicides to protect plants against MoT.

Lipopeptides (LPs) have been reported to be produced by distinctly different groups of bacteria and fungi. These compounds have been found to have antagonistic functions in interactions with various microorganisms, including plant fungal pathogens. Therefore, these natural compounds possess antifungal, antibacterial, antiviral, antitumor, anti-inflammatory, and immunosuppressive activities (Mondol et al., 2013). They consist of a linear or cyclic sequence of peptides incorporating an acyl chain, generally attached to the N-terminus, which has antimicrobial properties (Meena and Kanwar, 2015; Malmsten, 2016). The mode of action of LPs involves cell membrane perturbation (Oliveras et al., 2018). Members of the genus *Bacillus* are considered useful microbial factories for the broad-scale production of such bioactive molecules with antimicrobial action, including plant defense lipopeptide antibiotics such as surfactin, iturin, and fengycin are active suppressors of many plant pathogens (Wulff et al., 2002; Todorova and Kozhuharova, 2010; Roongsawang et al., 2011; Mondol et al., 2013). One of such pathogens include the rice blast fungus *M. oryzae* pathotype *Oryzae* (MoO) (Leelasuphakul et al., 2006). It has been reported that LPs are biodegradable and less toxic and have different biomedical applications (Singh and Cameotra, 2004; Tareq et al., 2014b). LPs are unlikely to induce an evolution of pathogen-resistant strains, as they do not target a particular receptor (Mondol et al., 2013). These compounds are, therefore, broadly regarded as potential alternatives to the growing issue of conventional antibiotic resistance, fungal infections, and life-threatening diseases.

Biological control through implementing antagonistic microorganisms or their secondary metabolites to plant pathogens is considered as an attractive alternative and a sustainable plant protection strategy (Kloepper et al., 1999; Shoda, 2000; Chan et al., 2003; Islam et al., 2005, 2011, 2016b). Some *Bacillus* spp. with antagonism against wheat blast fungus have been reported (Surovy et al., 2017). However, no report has so far been described regarding the antagonistic effects of secondary metabolites of any *Bacillus* spp. in the suppression of the wheat blast disease. In our laboratory, 150 natural compounds isolated from plants and microorganisms were screened against mycelial growth of wheat blast fungus MoT. Among them, five recently discovered linear non-cytotoxic LPs, namely, gageopeptide A–D (**1–4**) and gageotetrin B (**5**), isolated from the marine *Bacillus subtilis* strain 109GGC020 (Tareq et al., 2014a, b) displayed potent inhibitory effects against MoT *in vitro* and *in vivo*. To our knowledge, this is the first indication of the biological control of the fearsome wheat blast disease by the linear LPs from the *B. subtilis* strain 109GGC020. The specific objectives of this study were to (i) test the effects of five linear LPs (**1–5**) on mycelial growth of MoT; (ii) evaluate the effects of compounds **1–5** on conidiogenesis of wheat blast fungus; (iii) examine the effects of these compounds (**1–5**) on germination of conidia and their subsequent developmental transitions in sterilized water medium; and (iv) evaluate the effects of LPs (**1–5**) on suppression of wheat blast disease development in the detached leaves of wheat.

## Materials and Methods

### Chemicals

**FIGURE 1:**
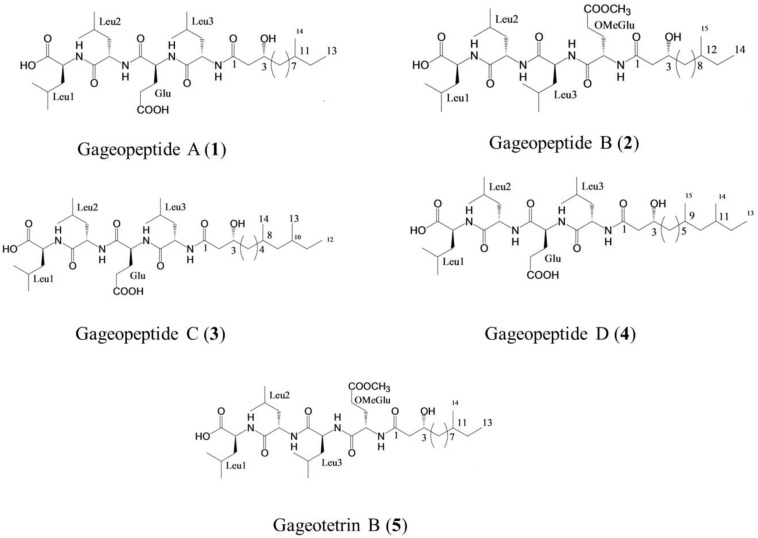
Structure of linear lipopeptides (**1–5**) isolated from *Bacillus subtilis* strain 109GGC020 tested toward *Magnaporthe oryzae Triticum* (MoT) (Tareq et al., 2014a, b).

Gageopeptide A (**1**), gageopeptide B (**2**), gageopeptide C (**3**), gageopeptide D (**4**), and gageotetrin B (**5**) (Figure 1) were isolated from *B. subtilis* strain 109GGC020 (Tareq et al., 2014a, b). Fungicide Nativo^®^ WG75 was purchased from Bayer Crop Science Ltd. Other compounds used in screening were either synthetic compounds purchased from the Sigma Aldrich or natural compounds available in the laboratory.

### Wheat Blast Strain, Media, and Plant Materials

The wheat blast strain BTJP 4 (**5**) was isolated from a blast-infected wheat spike cv. BARI Gom 24 (Prodip) collected from a severely infected field in the Jhenaidah district of Bangladesh in 2016. The strain was preserved on dried filter paper at 4°C until used in this study (Islam et al., 2016a). It was re-cultured on potato dextrose agar (PDA; 42 g/L) medium and incubated for 7–8 days at 25°C. After a small block of *Magnaporthe oryzae* pathotype *Triticum* isolate BTJP 4 (**5**) was placed in the aforementioned medium, the plate was incubated at 25°C (Islam et al., 2016a).

To induce sporulation, 10-day-old cultures grown on PDA medium were washed with 500 ml of deionized distilled water to remove the aerial mycelia and subsequently kept at room temperature (25–30°C) for 2–3 days (Urashima et al., 1993; Islam et al., 2016a). The conidial and mycelial suspension was filtered through two layers of cheese cloth. Conidia were harvested and mounted in water. Conidial germination was observed under microscope, and the numbers of germinated conidia were counted. A susceptible wheat cultivar BARI Gom 24 (Prodip) was used in disease suppression assay. Wheat leaves were detached from seedlings at the five-leaf stage for *in vivo* disease suppression bioassay (He et al., 2019).

### Inhibition of Mycelial Growth by Lipopeptides

The *in vitro* antifungal activity of the pure bioactive natural compounds and a commercial fungicide, Nativo^®^ WG75, was assessed on the basis of mycelial growth inhibition rate of MoT isolate BTJP 4 (**5**) by the modified Kirby–Bauer disk diffusion method (Bauer et al., 1966). A series of concentrations of each natural compound were prepared in ethyl acetate and fungicidal suspension of Nativo^®^ WG75 was prepared in distilled water. Filter-paper disks of 9-mm diameter (Sigma-Aldrich Co., St. Louis, MO, United States) were impregnated with bioactive compound solutions in concentrations ranging from 0.05 to 20 μg/disk. Paper disks were placed at one side of Petri dish (2 cm away from the edge) containing 20 ml of PDA. Five-millimeter-diameter size mycelial plugs from seven-day-old PDA cultures of MoT were placed at the opposite side of Petri dishes (diameter 9 cm) perpendicular to the paper disk. Petri dishes inoculated with fungal mycelia plugs against fungicide Nativo^®^ WG75 served as positive control, whereas fungal cultures without any treatment were used as negative control. Both positive and negative control treatments were replicated thrice. The minimum inhibitory concentration (MIC) of these LPs (**1–5**) was also determined. Antimicrobial activity was defined by the diameter of growth inhibition zone after 10 days. This experiment was replicated thrice for each concentration used. Plates were incubated at 25°C until fungal mycelia completely covered the agar surface in control plate. The radial growth of fungal colony was measured in centimeter with a meter ruler along two diagonal lines drawn on the reverse side of each plate. Data were recorded by measuring inhibition zone and mycelial growth of pathogen, and percent inhibition of radial growth (PIRG) (±standard error) (Riungu et al., 2008) was calculated from mean values as follows:

$$\text{PIRG}\left(\%\right)=\frac{\mathrm{Radial}\,\mathrm{growth}\,\mathrm{of}\,\mathrm{control}-\mathrm{Radial}\,\mathrm{growth}\,\mathrm{of}\,\mathrm{treatment}}{\mathrm{Radial}\,\mathrm{growth}\,\mathrm{of}\,\mathrm{control}}\times 100$$

Morphologies of hyphae in the vicinity of compounds were observed under a ZEISS Primo Star microscope at 40× and 100× magnification. Images of inhibition zone were taken with a digital camera (Canon DOS 700D), and images of hyphal morphologies were recorded using a microscope camera (ZEISS Axiocam ERc 5s).

### Inhibition of Conidiogenesis by Lipopeptides

Stock solutions of different LPs (**1–5**) were prepared in 100 μl of dimethyl sulfoxide (DMSO). Then 1,000 μg/ml of concentration of each compound was prepared in distilled water. The final concentration of DMSO in the working solution never exceeded 1% (v/v), which has no effect on mycelial growth and sporulation of the MoT. Fungicidal suspension of 10 ml of Nativo^®^ WG75 was prepared in distilled water (1,000 μg/ml), which was used as a positive control. For the observation of conidium formation (i.e., conidiogenesis), mycelia from the Petri plate of 5-day-old PDA culture of MoT fungus were washed out to reduce nutrient for the induction of conidiogenesis in the mycelia. Exactly 50 μl of each compound and Nativo^®^ WG75 at 1,000 μg/ml was applied on the 10-mm mycelial agar block of MoT fungus placed in Nunc multidish (Nunc), and only sterilized water with 1% DMSO was applied on the mycelial agar block of MoT fungus served as negative control. Plates with MoT mycelial agar blocks were incubated at 28°C with optimum humidity and lights. Conidium formation was observed under a light microscope (ZEISS Primo Star, Germany) at 10× magnification after 24 h, and the images were recorded with a microscope camera (ZEISS Axiocam ERc 5s). The experiment was repeated twice, and each time, it was performed in three replications.

### Inhibition of Conidial Germination and Developmental Transitions of Germinated Conidia by Lipopeptides

Stock solutions of different LPs (**1–5**) were prepared in small amounts of DMSO. Then 100 μg/ml concentration of each compound was prepared in distilled water where concentration of DMSO in final solution remained less than 1%. As a positive control, 100 μg/ml of Nativo^®^ WG75 solution was prepared with distilled water. The bioassays for conidial germination were carried out following protocols described earlier (Islam and von Tiedemann, 2011). Briefly, 100 μl of sample solution in appropriate concentration was directly added to 100 μl of conidial suspension (1 × 10^5^ conidia) of MoT taken in a well of a plant tissue culture multi-well plate to make a final volume of 200 μl at 50 μg/ml. Then it was quickly mixed with a glass rod and incubated at 25°C. The final concentration of DMSO in the conidial suspension never exceeded 1% (v/v), a condition that does not affect conidial germination or further developments of the germinated conidia. Control was treated with sterilized water with 1% DMSO. The multidishes containing conidia were incubated in a moisture chamber at 25°C for 6, 12, and 24 h in darkness, after which approximately 100 conidia from each of three replicates were examined under a light microscope (ZEISS Primo Star, Germany) at 100× magnification to determine the percentage of conidial germination and major morphological changes of the treated conidia, and the images were recorded with a microscope camera (ZEISS Axiocam ERc 5s). The experiment was repeated twice, and each time it was performed thrice. Time-course microscopic observation revealed that the treatments of conidia with tested compounds resulted in germination, no germination, normal/abnormal formation of the appressoria, alteration of the morphology of germ tubes, and/or mycelial growth. The percentage of conidial germination (±standard error) was calculated from mean values as follows: *CG*%=(*C*−*T*)/*C*× 100, where CG = conidial germination, *C* = percentage of germinated conidia in control, and *T* = percentage of germinated conidia in sample.

### Suppression of Wheat Blast Disease on Detached Leaves by Lipopeptides

Stock solutions of different LPs were prepared in small amounts of DMSO. Then 1,000 μg/ml of concentration of each compound was prepared in distilled water. The final concentration of DMSO never exceeded 1%, a condition that does not affect conidial germination and/or further development of the germinated conidia. Commonly used fungicide Nativo^®^ WG75 was used as a positive control. Sterilized water with 1% DMSO was used as negative control; 1,000 μg/ml of Nativo^®^ WG75 suspension was prepared by distilled water. Detached leaves of wheat at the five-leaf stage of seedlings were placed into dishes lined with moist absorbent paper, and each leaf was inoculated with three 20-μl droplets of the prepared compounds, and the leaves were allowed to absorb the compounds for 15 min. Subsequently, 1 × 10^5^ conidia of MoT were inoculated on the leaf surface. All dishes were incubated at 28°C with 100% relative humidity, first in darkness for 30 h and then in constant light for 2 days. The experiment was independently repeated five times. The diameter of wheat blast lesions was measured from 12 leaves per experiment. All data were statistically analyzed.

### Statistical Analysis, Experimental Design/Replications

Experiments for evaluating the biological activities of the pure compounds were carried out using a completely randomized design (CRD). Data were analyzed by one-way analysis of variance (ANOVA), and the mean values were separated by Tukey’s honestly significant difference (HSD) *post hoc* statistic. All the statistical analyses were performed using SPSS (IBM SPSS statistics 16, Georgia, United States) and Microsoft Office Excel 2010 program package. Mean value ± standard error of 3/5 replications were used in tables and figures.

## Results

### Inhibitory Effects of Lipopeptides on Mycelial Growth and Morphological Alterations of *Magnaporthe oryzae Triticum*

**FIGURE 2:**
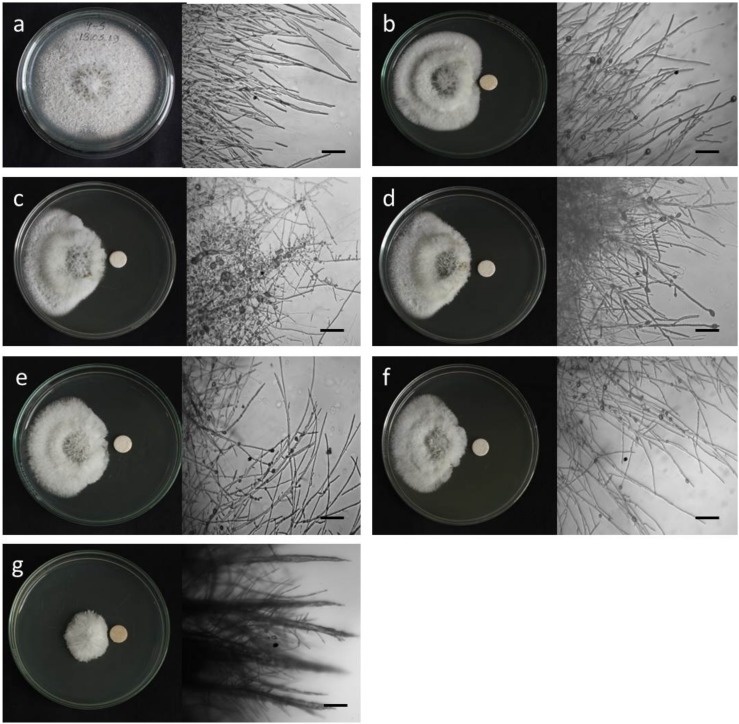
Macroscopic and microscopic view of *in vitro* antifungal activity of lipopeptides (**1–5**) against *Magnaporthe oryzae Triticum* (MoT) at 20 μg/disk. **(a)** Control, **(b)** gageopeptide A, **(c)** gageopeptide B, **(d)** gageopeptide C, **(e)** gageopeptide D, **(f)** gageotetrin B, and **(g)** Nativo^®^ WG75. Bar = 50 μm.

**FIGURE 3:**
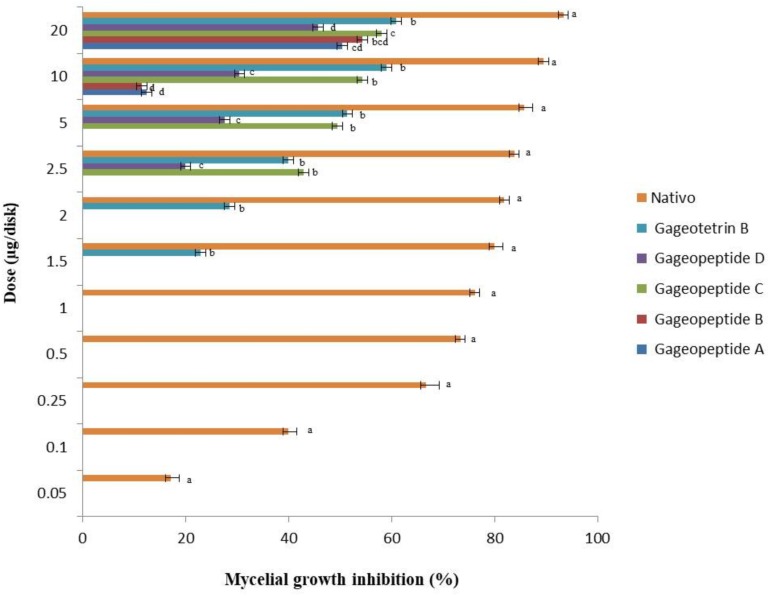
Inhibitory effects of bioactive lipopeptides (**1–5**) and a commercial fungicide Nativo^®^ WG75 on mycelial growth of *Magnaporthe oryzae Triticum* (MoT) in potato dextrose agar media. The data are the mean ± standard errors of three replications for each dose of the tested compound.

To see whether natural products inhibit MoT fungus, we tested a number of secondary metabolites from different plants and microorganisms. Out of 32 known natural compounds (Table 1) tested, the five-bioactive linear LPs (Figure 1) isolated from the *Bacillus subtilis* strain 109GGC020 displayed remarkable mycelial growth inhibitory capacity against MoT in PDA medium. Figure 2 shows the inhibition of mycelial growth of MoT by the tested LPs **1–5**. Among the five compounds, three compounds showed strong but varying levels of hyphal growth inhibitory activity against MoT isolate BTJP 4 (5). The highest mycelial growth inhibition of MoT isolate was observed by gageotetrin B (**5**), followed by gageopeptide C (**3**), gageopeptide B (**2**), gageopeptide A (**1**), and gageopeptide D (**4**). All compounds showed effectiveness on the tested pathogen ranging from 45.7 ± 1.6% to 60.9 ± 2.5% at 20 μg/disk (Figure 3). The activities of these LPs were significantly weaker than those of the commercial fungicide used in the field, with Nativo^®^ WG75 whose control percentage was 93.3 ± 0.9% (at 20 μg/disk).

**TABLE 1:** List of tested known compounds on mycelial growth of *M. oryzae Triticum* (MoT) fungus.

Name of compounds	Source	Activity against MoT
Gageopeptide A	*Bacillus subtilis*	Active
Gageopeptide B	*B. subtilis*	Active
Gageopeptide C	*B. subtilis*	Active
Gageopeptide D	*B. subtilis*	Active
Gageotetrin B	*B. subtilis*	Active
Gageotetrin C	*B. subtilis*	Inactive
Gageotetrin A	*B. subtilis*	Inactive
Gageostatin A	*B. subtilis*	Inactive
Gageostatin B	*B. subtilis*	Inactive
Gageostatin C	*B. subtilis*	Inactive
Macrolactin A	*Bacillus* sp.	Inactive
Macrolactin W	*Bacillus* sp.	Inactive
Macrolactin B	*Bacillus* sp.	Active
Staurosporine	*Streptomyces* sp.	Active
Feigrisolide C	*Streptomyces* sp.	Active
Bonactin	*Streptomyces* sp.	Active
Antimycin-A	*Streptomyces* sp.	Active
Natamycin	*Streptomyces* sp.	Inactive
Neomycin	*Streptomyces* sp.	Active
Oligomycin A + F	*Streptomyces* sp.	Active
Oligomycin F	*Streptomyces* sp.	Active
Oligomycin B	*Streptomyces* sp.	Active
Penicillin G	*Penicillium* sp.	Inactive
2,4-Diacetylphloroglucinol (DAPG)	*Pseudomonas fluorescens*	Inactive
Phloroglucinol (PG)	Synthetic	Inactive
Monoacetylphloroglucinol (MAPG)	Synthetic	Inactive
2,4-Dipropylphloroglucinol (DPPG)	Synthetic	Inactive
2,4,6-Triacetylphloroglucinol (TAPG 3)	Synthetic	Inactive
Chelerythrine chloride	*Chelidonium majus*	Active
2-Methyl-5-(8*Z*-heptadecenyl) resorcinol	*Ardisia sieboldii*	Inactive
5-(8*Z*-Heptadecenyl) resorcinol	*A. sieboldii*	Inactive
Ardisiaquinone A	*A. sieboldii*	Inactive

**FIGURE 4:**
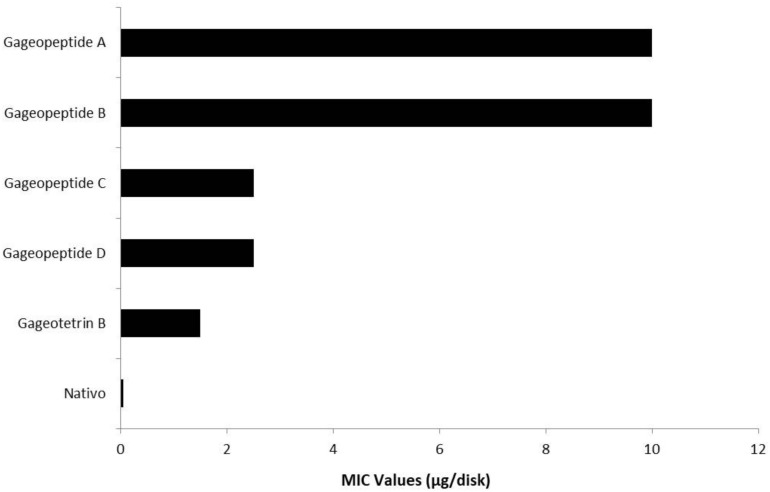
Minimum inhibitory concentration (MIC) values of bioactive lipopeptides (**1–5**) and a commercial fungicide Nativo^®^ WG75 on mycelial growth of *Magnaporthe oryzae Triticum* (MoT) in potato dextrose agar media.

All the different LPs inhibited mycelial growth of MoT but at varying strengths. The suppressive effect increased with the increase in concentration at 1.5–20 μg/disk (Figure 3). However, none of the LPs displayed any activity on MoT at concentration lower than 1.5 μg/disk. Compound **5** showed significantly higher inhibition of the pathogen at 20 μg/disk (60.9 ± 2.5%), followed by 10 μg/disk (59 ± 0.9%) and 5 μg/disk (51.4 ± 1.7%). The inhibition percentage of compounds **4** and **3** were 45.7 ± 1.6%, 30.5 ± 0.9%, 27.6 ± 0.9% and 58.1 ± 0.9%, 54.3 ± 1.6%, 49.5 ± 0.9%, respectively, at 20, 10, and 5 μg/disk. Again, compounds **2** and **1** showed 54.3 ± 3.3% and 50.5 ± 0.9% inhibitory percentage at 20 μg/disk. MICs of 10, 10, 2.5, 2.5, and 1.5 μg/disk (Figure 4) of these five natural compounds (**1**, **2**, **3**, **4**, and **5**) were also able to inhibit mycelial growth at 12.5 ± 1.8%, 11.5 ± 1.6%, 42.9 ± 1.6%, 20 ± 1.7%, and 22.9 ± 1.6%, respectively. Compared with the MICs of these five LPs, MIC of Nativo^®^ was 0.05 μg/disk (Figure 4).

Microscopic observations revealed that the untreated hyphae of *Magnaporthe oryzae Triticum* had regular and tubular growth with smooth, hyaline, branched, septate, plump, and intact hyphae (Figure 2a). On the contrary, the morphology of hyphae treated with the natural LPs was obviously different from that of the normal control hyphae. Unlike the tubular hyphae of the control group, remarkable increase in branch frequency per unit length of hyphae, reduced polar growth, and irregular swelling of the hyphal tips developed were observed when MoT was treated with compound **1** (Figure 2b). Compound **2** induced swelling of hyphal cells, hyper-branching, loss of apical growth, and swelling of hyphal tips (Figure 2c). Compound **3** commonly induced extensive branching, irregular growth, and swelling in MoT hyphae (Figure 2d). Compound **4** also caused disruption of normal polar growth of hyphae by inducing excessive branching and swelling of hyphal tips (Figure 2e). Compound **5** treated hyphae appeared as coarse, loss of radial growth, and irregular swelling on the tips (Figure 2f). In the presence of Nativo^®^ WG75, MoT hyphae showed crystal like appearance, irregular branching, and swelling with distorted grooves and corrugations (Figure 2g).

### Inhibitory Effects of Lipopeptides on Conidiogenesis of *Magnaporthe oryzae Triticum*

**FIGURE 5:**
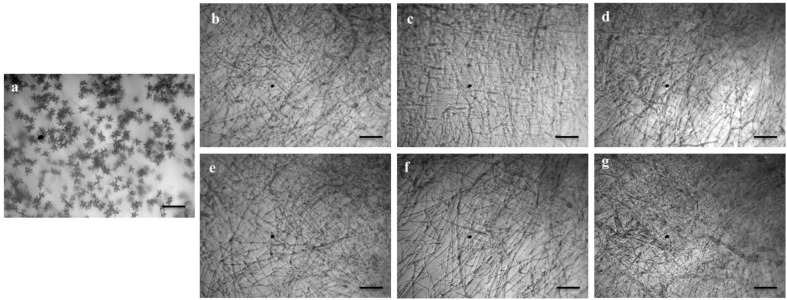
Effects of bioactive lipopeptides (**1–5**) on the suppression of conidiogenesis of *Magnaporthe oryzae Triticum* (MoT) in Nunc multidish at 1,000 μg/ml. **(a)** Control, **(b)** gageopeptide A, **(c)** gageopeptide B, **(d)** gageopeptide C, **(e)** gageopeptide D, **(f)** gageotetrin B, and **(g)** Nativo^®^ WG75. Bar = 50 μm.

We also tested whether the linear LPs (**1–5**) have an effect on the process of conidium formation. All the compounds inhibited conidiogenesis of MoT fungus. After 24 h of incubation, huge conidium formation was observed in the negative control mycelial agar block (Figure 5a). In case of positive control mycelial agar block, no (zero) conidium formation was observed (Figure 5g). Compared with negative control, conidiogenesis was completely inhibited by all the compounds (**1–5**) at 1,000 μg/ml of concentration (Figures 5b–f). However, microscopic observation showed the presence of broken hyphal tips and their slight growth on the surface of agar medium treated with LPs with no trace of conidiophore and conidial development.

### Inhibitory Effects of Lipopeptides on Conidial Germination of *Magnaporthe oryzae Triticum*

We used compound solution at 50 μg/ml to evaluate the inhibition of MoT conidial germination in a multi-well plate. Conidial germination percentage was recorded after 6 h of incubation (Table 2). In water, the conidial germination percentage was 100%, but in the presence of Nativo^®^ WG75, the conidial germination percentage was 0%. LPs (**1–5**) significantly suppressed the germination of MoT conidia. Among five compounds, gageopeptide D (**4**) showed the lowest conidial germination (25 ± 1.7%), followed by gageotetrin B (**5**) (45 ± 2.9%), gageopeptide B (**2**) (51 ± 2.1%), gageopeptide C (**3**) (52 ± 1.5%), and gageopeptide A (**1**) (61 ± 2.1%).

**TABLE 2:** Effects of linear lipopeptides from marine *Bacillus subtilis* strain 109GGC020 on germination of conidia and their subsequent developmental transitions of *Magnaporthe oryzae Triticum* (MoT) at the dose of 50 μg/ml *in vitro*.

Compound	Time (h)	Effects of natural lipopeptides on developmental transitions of conidia of wheat blast fungus *M. oryzae Triticum* (MoT)	
		Germinated conidia (% ± SE^a^)	Major morphological change/developmental transitions in the treated conidia
Water	0	0 ± 0f	No germination
	6	100 ± 0a	Germinated with normal germ tube and normal appressoria developed
	12	100 ± 0a	Mycelial growth took place
	24	100 ± 0a	Huge mycelial growth took place
Gageopeptide A (**1**)	0	0 ± 0^f^	No germination
	6	61 ± 2.1b	Germinated with a short germ tube
	12	31 ± 1.7c	Normal germ tube
	24	27 ± 1.5c	Normal appressoria formed; no mycelial growth took place
Gageopeptide B (**2**)	0	0 ± 0^f^	No germination
	6	51 ± 2.1cd	Germinated with a short germ tube
	12	51 ± 2.1b	Deformed germ tube (swollen)
	24	0 ± 0f	No appressoria formed; no mycelial growth took place
Gageopeptide C (**3**)	0	0 ± 0^f^	No germination
	6	52 ± 1.5c	Germinated with a short germ tube
	12	10 ± 1.5e	Deformed germ tube (swollen)
	24	41 ± 0.7b	30% Normal appressoria and 11% abnormal appressoria (low melanization) formed but no mycelial growth took place
Gageopeptide D (**4**)	0	0 ± 0^f^	No germination
	6	25 ± 1.7e	Germinated with a short germ tube
	12	25 ± 1.7d	Deformed germ tube (swollen)
	24	0 ± 0f	No appressoria formed; no mycelial growth took place
Gageotetrin B (**5**)	0	0 ± 0^f^	No germination
	6	45 ± 2.9d	Germinated with a short germ tube
	12	41 ± 3.8c	Deformed germ tube (swollen)
	24	4 ± 1d	Abnormal appressoria formed; no mycelial growth took place
Nativo^®^ WG75	0	0 ± 0f	No germination
	6	0 ± 0f	No germination
	12	0 ± 0f	No germination
	24	0 ± 0f	No germination

*^*a*^Data presented here are mean value ± SE of three replications in each compound. Means within a column followed by the same letter(s) are not significantly different as assessed by Tukey’s honestly significance difference (HSD) *post hoc* (*p* ≤ 0.05).*

### Effects of Lipopeptides on Morphological Changes of the Treated Conidia of *Magnaporthe oryzae Triticum*

**FIGURE 6:**
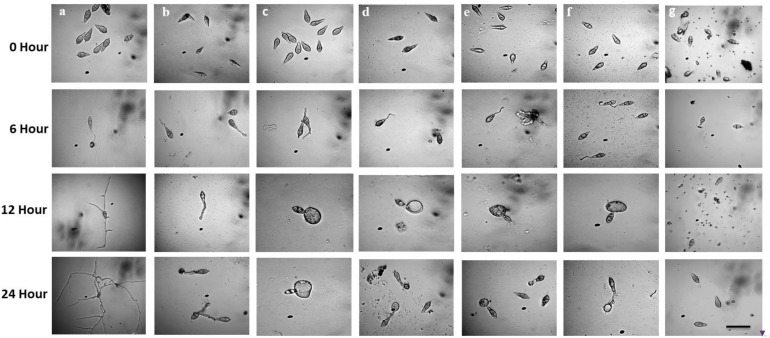
Time-course changes in germination of *Magnaporthe oryzae Triticum* (MoT) conidia and their developmental transitions in the presence of linear lipopeptides (**1–5**). Dose of lipopeptides was 50 μg/ml. **(a)** Control, **(b)** gageopeptide A, **(c)** gageopeptide B, **(d)** gageopeptide C, **(e)** gageopeptide D, **(f)** gageotetrin B, and **(g)** Nativo^®^ WG75. Bar = 10 μm.

Following incubation at 25°C for 24 h in darkness, 100% conidial germination with normal germ tube development and mycelial growth was observed in treatments with water after 6, 12, and 24 h (Table 2 and Figure 6a), whereas the effects of the five linear LPs on the further development of the conidia varied with time, and abnormal morphological changes were also observed by some compounds at 50 μg/ml. In the presence of gageopeptide B (**2**) and gageopeptide D (**4**), 51 ± 2.1% and 25 ± 1.7% conidial germination took place after 6 h but induced 51 ± 2.1% and 25 ± 1.7% abnormally swollen germ tubes with no appressorial formation and mycelial growth after 12 and 24 h, respectively (Table 2 and Figures 6c,e). In case of gageotetrin B (**5**), 45 ± 2.9% conidia germinated after 6 h; 41 ± 3.8% conidial germ tubes were deformed (swollen) after 12 h, and the rest produced abnormal appressoria after 24 h (Table 2 and Figure 6f). Gageopeptide A (**1**) induced 61 ± 2.1% conidial germination after 6 h, with 31 ± 1.7% normal germ tube and 27 ± 1.5% normal appressorial formation after 12 and 24 h but inhibited 100% mycelial growth (Table 2 and Figure 6b). In the presence of gageopeptide C (**3**), 52 ± 1.5% conidia were germinated after 6 h. Among them, 10 ± 1.5% germ tubes became swollen after 12 h. At 24 h after treatment, 30 ± 0.7% conidia produced normal appressoria, whereas 11 ± 0.7% produced abnormal appressoria (low melanization) without progressing to mycelial growth (Table 2 and Figure 6d). Conidial lysis was not found in any treatment. The results showed that the five LPs cause significant malformation of germ tubes and inhibit appressorial formation and mycelial growth of *M. oryzae Triticum.* In case of Nativo^®^ WG75, no conidial malformation was observed (Figure 6g).

### Suppression of Wheat Blast Disease by Lipopeptides From *Bacillus subtilis*

**FIGURE 7:**
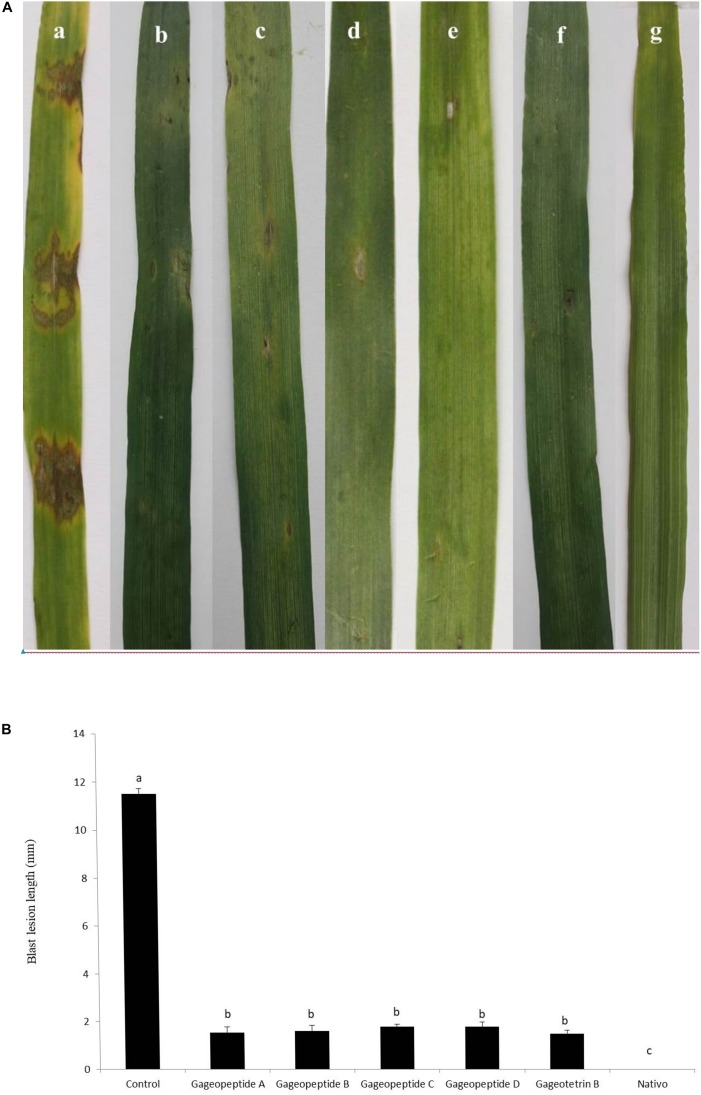
Effects of bioactive lipopeptides (**1–5**) on the suppression of lesion formation in detached wheat leaves by *Magnaporthe oryzae Triticum* at 1,000 μg/ml. **(A)** Blast lesion symptoms on treated and untreated wheat leaves: **(a)** control, **(b)** gageopeptide A, **(c)** gageopeptide B, **(d)** gageopeptide C, **(e)** gageopeptide D, **(f)** gageotetrin B, and **(g)** Nativo^®^ WG75. **(B)** Diameter of lesions were measured. Bars represent ± standard error. Means followed by the same letters are not significantly different at the 5% level according to Tukey’s honestly significance difference (HSD) *post hoc* statistic.

Application of compounds **1–5** at 1,000 μg/ml significantly reduced wheat blast symptoms in detached leaves inoculated with the conidia of MoT. The average lesion length recorded in the leaves treated with compounds **1–5** were 1.5 ± 0.2, 1.6 ± 0.2, 1.8 ± 0.1, 1.8 ± 0.1, and 1.5 ± 0.1 mm (Figures 7A,B). These results indicate that all natural compounds significantly inhibited wheat blast development on detached wheat leaves. On the other hand, in inoculated negative control plants having water treatments, typical blast disease spots were 11.5 ± 0.2 mm in length, but no visible disease symptoms were observed on the leaves when treated with Nativo^®^ WG75 at 1,000 μg/ml (Figures 7A,B).

## Discussion

In this study, we found that five linear LPs, namely, gageopeptide A (**1**), gageopeptide B (**2**), gageopeptide C (**3**), gageopeptide D (**4**), and gageotetrin B (**5**), isolated from the marine bacterium, *Bacillus subtilis* strain 109GGC020, showed strong antifungal activities against a destructive wheat blast pathogen, *Magnaporthe oryzae Triticum*. Bioassay results revealed that all the compounds remarkably inhibited mycelial growth as well as conidiogenesis, germination, and developmental transition of the germinated conidia and further suppressed wheat blast disease in detached wheat leaves. Therefore, these results suggest that inhibition of mycelial growth and conidial germination by these LPs correlates with the suppression of wheat blast disease upon leaf inoculation. Inhibition of hyphal growth, conidiogenesis, and conidial germination of different fungi including rice blast fungus by several natural secondary metabolites on *in vitro* bioassay have been reported by many earlier investigators (Romero et al., 2007; Kopecká et al., 2010; Tang et al., 2014; Gond et al., 2015; Liao et al., 2016; Zhang and Sun, 2018; Rodríguez-Chávez et al., 2019). This is the first report of controlling the destructive wheat blast fungus using secondary metabolites (linear LPs **1–5**) isolated from *B. subtilis*. In addition, LPs are unlikely to induce pathogen-resistant strains to evolve, as they do not target a particular receptor (Mondol et al., 2013). Despite the outstanding biological properties of LPs, so far, only a few studies have focused on their development as plant protection agents. Most of the reports focused on cyclic LPs synthesized by *B. subtilis* strains because of their diversity of biological properties, including the ability to respond to plant defense (Meena and Kanwar, 2015). However, this is also a first report on suppression of wheat blast fungus MoT by some natural linear LPs from *B. subtilis*.

One of the striking findings in this present study is the swelling behavior of these LPs on the hyphae and on the hyphal tips of MoT (Figures 2b–f). We tested a range of concentrations ranging from 0.05 to 20 μg/disk. Swelling increased with increasing concentrations. Swelling in the fungal hyphae and on the tips of hyphae has been reported earlier by cyclic LPs fengycin (Gond et al., 2015; Liao et al., 2016; Zhang and Sun, 2018) and tensin (Nielsen et al., 2000). According to a recent report of Omoboye et al. (2019), some newly purified cyclic LPs, bananamides D–G from *Pseudomonas* sp. COW3, significantly inhibited mycelial growth of *Pseudomonas oryzae* and *Pseudomonas myriotylum* followed by extensive branching, hyphal leakage, and vacuolization of the mycelia. So far, this is a first report on swollen-like predatory behavior of some linear LPs toward a destructive microorganism. Morphological alterations including excessive branching and hyphal swelling of an oomycete phytopathogen *Aphanomyces cochlioides* by xanthobaccin A from a rhizoplane bacterium *Lysobacter* sp. SB-K88 or phloroglucinols from *Pseudomonas fluorescens* have been reported (Islam et al., 2005; Islam, 2008, 2010; Islam and Fukushi, 2010). A further investigation is needed to understand the mode of action of the linear LPs **1–5** toward the worrisome phytopathogen MoT.

Conidia are the propagules of infecting plants by most of the phytopathogenic fungi (Ohara, 2004), and the process by which these structures are produced by fungal cells is termed conidiogenesis (Kopecká et al., 2010). Disruption of conidiogenesis and conidial germination eliminates the possibility of infection by a fungal phytopathogen. Another interesting finding of this study is that all the five linear LPs (**1–5**) remarkably inhibited conidiogenesis (Figure 5), germination, and developmental transition of the conidia (Table 2 and Figure 6). Bioassay results revealed that prior treatment of wheat leaves with these LPs at 1,000 μg/ml, resulted in the leaves being less attractive to MoT conidia. Another most important phenomenon observed in the MoT conidia interacting with some linear LPs was the dynamics of the swollen behavior in the conidial germ tube (Figures 6c–f). A similar phenomenon was observed by Liao et al. (2016), who reported that the antifungal cyclic lipopeptide fengycin inhibits conidial germination of *M. oryzae Oryzae* (MoO) by deforming the conidia and germ tube (swollen) and halting appressoria formation. After exposure to a cyclic lipopeptide mixture containing iturins and fengycins, the conidia of *Penicillium expansum* also showed more than 90% of swelling and germination reduction (Rodríguez-Chávez et al., 2019). Some LPs induced abnormal appressoria (low melanization), which inhibited the infection process of MoT fungus, as appressorium melanization is required for pathogenicity of *M. oryzae* (Wang et al., 2005; Wilson and Talbot, 2009). This study for the first time demonstrated that the five novel linear LPs inhibited conidiogenesis, germination, and appressoria formation of the conidia of MoT. These compounds might affect the expression of genes upstream from melanin synthesis. Future studies should therefore also focus on the effects of these natural bioactive compounds on the expression of conidial germination and appressorium formation-related genes in MoT.

In earlier studies, these linear LPs (**1–5**) showed antimicrobial activities against several fungal and oomycetal phytopathogens such as *Rhizoctonia solani*, *Botrytis cinerea*, *Colletotrichum acutatum*, and *Phytophthora capsici* but did not exhibit any cytotoxicity (GI50 > 25–30 μM) against human cancer cell lines (Tareq et al., 2014a, b). Although structure–activity relationships were not established in case of these novel metabolites, further study is needed to derivatize these compounds by changing their side chains to elucidate the structure–activity relationship of these LPs (Figure 1). On the other hand, structural features of these compounds might be linked with their variable inhibitory activities against the MoT (Figure 1). All the compounds have acyl chain with a Leu-rich peptide backbone and a 3-hydroxy fatty acid (Tareq et al., 2014a, b). Among them, compound **5** and compound **2** have an OMeGlu amino acid residue in the peptide unit and a new 3-hydroxy fatty acid (HDDA) in the fatty acid chain. The acyl chain is essential for the biological activity of LPs. The acylation of a peptide sequence is considered as a means of increasing membrane affinity and therefore essential for antimicrobial activity (Malmsten, 2016). On the other hand, 3-hydroxy fatty acid itself also has strong antifungal properties (Sjogren et al., 2003). A further study is needed to establish the precise structure–activity relationships of these LPs, which would enable the synthesis of more active peptide, which is an effective agrochemical against MoT.

The morphological alterations shown in MoT by these LPs are also related to some potent, cell-permeable, and selective antifungal secondary metabolites, such as staurosporine, chelerythrine chloride, polyoxin A, polyoxin B, and polyoxin D. Staurosporine and chelerythrine chloride are well-known protein kinase C inhibitor, which caused swelling of hyphal tips and subapical regions and inhibited spore germination and normal appressoria formation of plant pathogenic fungi through the prevention of ATP binding to the kinase (Agae and Magae, 1993; Penn et al., 2015; Wei et al., 2017; Sugahara et al., 2019). Polyoxins are known as cell wall biosynthesis inhibitors, which inhibit germ tube formation and cause hyphal tip swelling (Isono et al., 1965; Endo et al., 1970; Copping and Duke, 2007; Barka et al., 2016).

A hallmark of the findings of this research is that application of all the linear LPs (**1–5**) remarkably suppressed blast disease in detached wheat leaves (Figure 7). During this study, wheat leaves previously treated with the LPs had smaller lesion lengths than had the untreated control (Figure 7). Most of the lesions were small and brown, having specks of pinhead size (scale 1) to small, roundish to slightly elongated, gray spots about 1–2 mm in diameter (scale 3). On the other hand, the untreated control leaves had typical blast lesions infecting 26–50% of the leaf area (scale 7), as per 9 scale of blast disease assessment given by the standard evaluation system of IRRI (1996). But in the presence of Nativo^®^ WG75, no visible blast lesions were observed. Nativo^®^ WG75 is a systemic broad-spectrum commercial fungicide, which we used as a positive control. The antifungal activities of these LPs on the suppression of MoT fungus are quite similar to the antifungal activities of this fungicide. Nativo^®^ WG75 contains two active ingredients, tebuconazole and trifloxystrobin. Tebuconazole is a systemic triazole fungicide that is known as demethylation inhibitor (DMI). It interferes with sterol biosynthesis in fungal cell walls and inhibits the spore germination and further growth of the fungus (Pring, 1984). Trifloxystrobin is a strobilurin fungicide, which interfered with respiration in plant pathogenic fungi by inhibiting electron transfer in mitochondria, disrupting metabolism, and preventing growth of the target fungi (Sauter et al., 1999). The results of this study are also comparable with activities of other commercially available fungicides like blasticidin-S, kasugamycin, streptomycin, oligomycin A, and tricyclazole, which showed strong antifungal activities against several phytopathogenic fungi including rice blast fungus MoO (Yamaguchi, 1982; Woloshuk et al., 1983; Kim et al., 1999; Magar et al., 2015). For the first time, this study demonstrated the disease suppression of wheat blast by some novel secondary metabolites from the marine *B. subtilis*. The common mode of action of these fungicides is to inhibit protein synthesis, cell wall biosynthesis, and melanin biosynthesis, which consequently resulted in suppressing fungal mycelial growth, spore germination, and normal appressoria formation.

The experimental findings of this work suggest that the interactions between LPs and MoT fungus are likely unknown multifaceted processes, which might have mediated likely by affecting the membrane integrity and organelle dysfunction, thereby resulting in cell death of *M. oryzae Triticum*. The combined effects evoked complex responses that lead to a series of intracellular events and ultimately death of the pathogen. A further investigation is needed to elucidate the mode of action of inhibitory effects of the novel LPs on wheat blast fungus.

Induced resistance as a mechanism of biological control by lipopeptides has been also reported earlier (Ongena et al., 2007). The surge in resistance to fungicides among pathogenic microorganisms is an emerging topic in agriculture nowadays. The non-rational use of fungicides with site-specific modes of action, such as the strobilurin (QoI) and triazole, has resulted in widespread distribution of some resistant mutant population in MoT (Dorigan et al., 2019). The most alarming antifungal resistance issue of conventional fungicides leads to search for novel safe antifungal agents to protect wheat plants from this threatening phytopathogenic fungus. Although novel LPs has lower bioactivity than has the commercial fungicide Nativo^®^, the inhibitory capacity of these compounds against MoT should enable their consideration for synthesis as candidate agrochemicals with novel mode of action against the destructive wheat blast fungus.

In conclusion, our results, for the first time, demonstrated that the five linear LPs from the *B. subtilis* strain 109GGC020 inhibited mycelial growth and asexual development of MoT fungus and suppressed wheat blast disease in detached wheat leaves. Field evaluation of these linear LPs is needed to judge these metabolites as effective fungicides against wheat blast disease. A further study is also necessary to understand the mode of action and structure–activity relationships of these novel bioactive LPs (**1–5**) toward the notorious cereal killer, *M. oryzae Triticum*.

## Data Availability Statement

All datasets generated for this study are included in the article/supplementary material.

## Author Contributions

TI conceived the idea and coordinated, critically edited, and revised the manuscript. HS and FT isolated and determined the structures of the lipopeptides and revised the manuscript. MC designed and executed the experiments, analyzed the data, and wrote the manuscript. DG designed and executed the experiments. NM designed the experiments and prepared the graphs and pictures.

## Conflict of Interest

The authors declare that the research was conducted in the absence of any commercial or financial relationships that could be construed as a potential conflict of interest.

## Footnotes

1 http://faostat.fao.org
